# Intrinsic Aqueous Solubility: Mechanistically Transparent Data-Driven Modeling of Drug Substances

**DOI:** 10.3390/pharmaceutics14102248

**Published:** 2022-10-21

**Authors:** Mare Oja, Sulev Sild, Geven Piir, Uko Maran

**Affiliations:** Institute of Chemistry, University of Tartu, Ravila 14A, 50411 Tartu, Estonia

**Keywords:** solubility, drug substances, QSAR, QSPR, fit-for-purpose training set, multiple linear regression, consensus model

## Abstract

Intrinsic aqueous solubility is a foundational property for understanding the chemical, technological, pharmaceutical, and environmental behavior of drug substances. Despite years of solubility research, molecular structure-based prediction of the intrinsic aqueous solubility of drug substances is still under active investigation. This paper describes the authors’ systematic data-driven modelling in which two fit-for-purpose training data sets for intrinsic aqueous solubility were collected and curated, and three quantitative structure–property relationships were derived to make predictions for the most recent solubility challenge. All three models perform well individually, while being mechanistically transparent and easy to understand. Molecular descriptors involved in the models are related to the following key steps in the solubility process: dissociation of the molecule from the crystal, formation of a cavity in the solvent, and insertion of the molecule into the solvent. A consensus modeling approach with these models remarkably improved prediction capability and reduced the number of strong outliers by more than two times. The performance and outliers of the second solubility challenge predictions were analyzed retrospectively. All developed models have been published in the QsarDB.org repository according to FAIR principles and can be used without restrictions for exploring, downloading, and making predictions.

## 1. Introduction

Intrinsic aqueous solubility ($S_{0}$) is an essential property that describes the solubility of a compound in an uncharged state. It is different from aqueous solubility because it is independent of pH and, together with the dissociation constant ($pK_{a}$), can be used to calculate the solubility at different pH values. This makes it an important physicochemical parameter for characterizing in vivo dissolution to understand processes in pharmaceutical science and drug discovery [1,2,3]. An example of use is the detection of suitable biowaivers for in vivo bioavailability and bioequivalence studies, where the predictive models of aqueous solubility are alternative to the experimental measurements in the biopharmaceutical classification system (BCS) [4]. Another example is the application of accurate prediction models to eliminate drug substance candidates that may precipitate or aggregate during high-throughput in vitro experiments in order to avoid false results from biological activity measurements [5,6]. Thus, the calculation (prediction) of intrinsic aqueous solubility is important both for the mechanistic understanding of the solubility processes and for reducing resources and saving time, for example in the pharmaceutical industry [7,8].

Surprisingly, due to the diversity and inconsistency of aqueous solubility data, the availability of intrinsic aqueous solubility data in the literature and databases is very limited. Solubility data are represented using diverse terms such as solubility in water, intrinsic solubility, solubility in a single buffered solution, turbidimetric solubility, kinetic solubility, etc. [9]. Individual solubility values published in the literature and databases often lack details of experimental measurement protocols. Most of published data describes the solubility in water as measured in distilled water, where the property depends on the type and form of the compound [9]. This is not a significant problem for highly soluble and neutral compounds because their aqueous solubility values are the same or very close to the intrinsic aqueous solubility, which describes the solubility of uncharged compounds. However, this is a problem in the case of salts and ionizable compounds, where the water solubility can be significantly different from the intrinsic aqueous solubility as they correspond to different ionization states of the compound. Note that approximately 85% of drug substances are ionizable compounds [10]. Therefore, their solubility should be evaluated in terms of intrinsic aqueous solubility because it does not depend on pH and is experimentally reproducible compared to solubility in water. As already mentioned, intrinsic aqueous solubility together with the dissociation constant ($pK_{a}$) can be used to calculate the solubility at different pH values.

Over decades of research, various data-driven [11,12] and physics-based [13,14] computational approaches have been proposed for estimating aqueous solubility from the chemical structure. Among them, more common are the data-driven quantitative structure–property relationships (QSPRs) [2,11,12,15,16]. Many QSPRs have been developed for water solubility but not for intrinsic aqueous solubility [17]. The solubility of drug substances is difficult to predict for a variety of reasons; one of the main issues is the poor consistency and reliability of solubility data for drug substances, which significantly reduces confidence in solubility prediction models. This is because the experimental determination of solubility is not a trivial task, which may significantly affect solubility in the test protocol used [18], including the polymorphism of crystals, differences in solubility of amorphous and crystalline analogs, salts and neutral compounds, hydrates, and anhydrous compounds [9,19]. Intrinsic solubility values can be calculated from the solubility in water or solubility in buffered solution values; however, it requires clearly documented test protocols and information about solution pH, which are usually unavailable [20]. The proper characterization of the structural features related to the solubility of chemical compounds is equally important for developing QSPRs. For example, the molecular descriptors calculated from the chemical structure do not describe the packing of the crystals or the solid state of the compounds. [21,22]

The first Solubility Challenge [19] was held in 2008 to address the issues related to the intrinsic aqueous solubility prediction of drug substances. Within this challenge, the QSPR modeling community was provided with a high-quality training set of 100 drug substance-like compounds to derive QSPR models for predicting the intrinsic aqueous solubility of an external test set of 32 drug substance-like compounds. The call received 99 contributions. The most interesting outcome of the Solubility Challenge 2008 was that all methods for deriving prediction models worked similarly well, and the best method for predicting intrinsic aqueous solubility could not be identified [18]. Ten years later, in 2019, the second Solubility Challenge was announced, but on a completely different basis [23]. This time no training data was provided to the QSPR modeling community for deriving the models, instead participants had to predict the intrinsic aqueous solubility of the two curated data series from the shake-flask data measured in several laboratories. The two data series differed in their interlaboratory standard deviation (see Section 2.1 for details). Participants had to use their existing QSPR model(s) or create a new training set, and the use of test set compounds for training was prohibited. The Solubility Challenge 2019 (SC2019) was joined by twenty QSPR development teams, who provided predictions of 37 different models, including predictions from the current authors [24].

At the time of the writing this manuscript, two studies have been published describing the modelling efforts by SC2019 participants who provided predictions for the challenge [25,26]. Mitchell [25] collected intrinsic aqueous solubility values for 153 compounds from four literature sources and developed three models using the following methods: Random Forest, Extra Trees, and Vox Machinarum (consensus of three machine learning models). The predictions showed that the tight test set was predicted with the highest accuracy using the Extra Trees method and the loose test set with Vox Machinarum and Random Forest (see the meaning of tight and loose test sets in the Section 2.1). Lovrić et al. [26] assembled a data set of 829 drug substance-like compounds with intrinsic aqueous solubility values and modeled this with an array of machine learning methods (Random Forests, Light Gradient Boosting Machine (LightGBM), partial least squares, and least absolute shrinkage and selection operator (LASSO)). From the resulting models, predictions from three LightGBM models were submitted to SC2019, which were among the top performers in predicting the intrinsic aqueous solubility of both test sets [24].

Tight and loose test sets from SC2019 have been independently used four times to test new and existing models [20,27,28,29]. Avdeef [20] has collected aqueous solubility data from 1325 literature sources (multi-source compilations, single-source measurements for many compounds, and miscellaneous primary sources) and curated and analyzed this data to obtain 6355 intrinsic aqueous solubility values for 3014 different chemical compounds. The model was developed using the random forest regression (RFR) approach and it gave the best prediction for the SC2019 test data sets (see Section 3.6). Falcón-Cano et al. derived a recursive random forest model prior to SC2019 to predict water solubility [30] and the SC2019 data sets allowed them to test the predictive power of their model [27]. They found that despite their training set having inconsistencies related to pH, solid form, and temperature, the developed model had comparable prediction capability to the top-ranked models in SC2019. Tosca et al. [28] developed an artificial neural networks QSPR model for intrinsic aqueous solubility and predicted both test sets with a sufficient prediction accuracy. Francoeur and Koes [29] used molecule attention transformer (MAT) architecture to develop a model (called SolTranNet) for data from AqSolDB. The model provided lower prediction capability for the SC2019 test sets compared to the models developed for the challenge.

The main scientific objective of the present research was to find an optimal solution for the development of QSPR models specifically for the intrinsic aqueous solubility of drug substances that are interpretable and easy to use. The discussions in preparing for the study led to the hypothesis that by constructing training data on the principle of fit-for-purpose it is possible to derive models that are both mechanistically transparent and accurate. The fit-for-purpose principle has two objectives: the training data set must consist of drug substances, and it must contain intrinsic aqueous solubility values with the same experimental accuracy as the test data sets of SC2019. The following chapters provide an overview of the choices made in constructing QSPR workflows, present the results, and compare them with other prediction results presented in SC2019. The application of a consensus modeling approach was further explored so that the predictions of the derived QSPR models could complement each other. All developed models have been made openly available according to FAIR principles and can be used without restrictions for exploring and making predictions.

## 2. Data & Methodology

Drug substances form a distinct and versatile chemical space, so there was no need to involve other non-drug substance-like compounds. It was assumed that most SC2019 participants would try to apply predictions from models derived using complex machine learning and artificial intelligence methods. Therefore, it was decided to provide predictions based on multilinear regression models derived from different sets of molecular descriptors using different descriptor selection approaches. The data sets, sources of molecular descriptors, descriptor selection methods, and model development environments are described in the following subchapters and summarized in Table 1.

### 2.1. Solubility Challenge 2019 Data—The Test Sets

SC2019 presented drug substances that formed two test sets with different interlaboratory standard deviations (SD) for intrinsic aqueous solubility ($logS_{0}$) [23]. The first test data set comprised 100 drug substances with a low SD between at least three laboratories (less than 0.5 log units, on average ~0.17), i.e., they were with high consensus and the set was named the ‘tight test set’. The second test set with 32 drug substances had a high interlaboratory SD, higher than 0.5 log units (on average ~0.62), i.e., the data points were with low consensus, and the set was named ‘loose test set’. Experimental values for the test sets were not available during SC2019 [23]. Their solubility values were published after the challenge [20].

### 2.2. Intrinsic Aqueous Solubility Data for Training and Validation

To derive models, two different fit-for-purpose sets of data were compiled so that their precision and applicability domain corresponded to the SC2019 test data sets. Although thousands of data points with solubility data have been compiled in the literature and in various databases (see for example solubility data compilation [31,32]), it was a challenge to collect a representative training set for intrinsic aqueous solubility only. As was explained in the introduction, the solubility values published in the literature and databases have very different experimental backgrounds and are very difficult or impossible to verify. Therefore, it was decided to limit only to those literature sources for which it was certain that intrinsic aqueous solubility values were included.

Training data set 1 and the corresponding validation data set were based on a compilation [33] containing 123 drug substances. The intrinsic aqueous solubility for these compounds was calculated from the solubility data measured by the CheqSol method, and the average inter-laboratory error was 0.15 log units, which was lower than the standard shake-flask method. Compounds that were also present in the SC2019 test sets were removed from the training sets. Since the reference values for the SC2019 test set compounds were unknown at the model development stage, the overlapping compounds became a part of the validation set. As a result, training data set 1 consisted of 81 compounds and the corresponding validation data set of 42 compounds. To clarify, overlapping compounds in the validation and SC2019 test data sets had different intrinsic aqueous solubility values because SC2019 provided averaged values from multiple literature sources. Training set 1 was more limited in its property range due to the lack of compounds in the open literature with low experimental error for very high and very low solubility data.

Training data set 2 and the corresponding validation data set consisted of drug substances with a wider solubility range and more structural diversity; data from twelve additional literature sources [18,19,34,35,36,37,38,39,40,41,42,43] were added to the previous data set [33]. Due to the large number of new sources, data curation and pretreatment were carried out. The intrinsic aqueous solubility data were converted to log molar unit ($logS_{0}$ [M]) because they were in different units. Median $logS_{0}$ values were calculated for compounds with multiple $logS_{0}$ values. The outcome of data curation and pretreatment steps yielded 436 unique compounds. Again, compounds present in the SC2019 test sets were excluded from the training set. This resulted in a training data set 2 of 346 compounds and a corresponding validation set of 90 compounds. It should be noted here that most of the intrinsic aqueous solubility values from the above references are also aggregate values because they were collected from several literature sources (read more about the curation of literature data here [44]).

### 2.3. Descriptor Calculation and Modeling Methods

The chemical structures in the data sets described above were characterized with molecular descriptors from different software packages. Three multi-linear regression (MLR) models were derived for the intrinsic aqueous solubility using different approaches to descriptor selection. An overview of molecular descriptor sources, descriptor selection methods, and model development environments is summarized in Table 1. The molecules were represented as 2D SMILES, and only 1D and 2D descriptors were calculated. Standardizer (version 19.10.0) [45] was used to unify the representation of aromaticity and nitro groups in SMILES structures.

The first model (M1) was for a small high-consensus training data set 1. For this, 4885 descriptors were calculated with Dragon software (version 6.0.40) [46]. The model was developed using the best multiple linear regression (BMLR) method [47,48] in CODESSA Pro (version 1.0) [49,50], where descriptors were selected based on a stepwise forward selection algorithm that starts with the elimination of insignificant descriptors and descriptors with missing values, followed by the selection of best models based on statistical and non-collinearity criteria of the selected descriptors. Descriptors were analyzed for their mechanistical significance to the solubility process.

The second model (M2) was developed for the larger and more structurally diverse training data set 2 where 238 molecular descriptors were calculated with the open-source cheminformatics toolkit RDKit (version 2016.03.05) [51]. The calculated molecular descriptors were filtered by removing descriptors with missing values or zero variance. For the development of the MLR model, the orthogonal matching pursuit (OMP) algorithm [52] from scikit-learn (version 0.18) [53,54] was used for the selection of molecular descriptors. The number of molecular descriptors for M2 was selected with an iterative approach where the OMP algorithm was used to build models with different number of descriptors. The optimal number of descriptors was found when increasing the descriptor count in the model resulted only in a minor improvement.

The third model (M3) was also based on training data set 2 and the pool of 1786 molecular descriptors comprised of solubility calculated with XLOGS (version 1.0) [55,56] and molecular descriptors from PaDEL-Descriptor (version 2.21) [57,58] software. The selection of molecular descriptors was carried out with a multistep procedure, where the Random Forest (RF) [59] algorithm was used for the preselection of significant descriptors, followed by an exhaustive search to find the best combination with up to three descriptors for the optimal MLR model. At first, descriptors with missing values or zero variance, highly correlated (R > 0.9) descriptors, and different octanol/water partition coefficient descriptors were removed. The second step involved building 100 preliminary models using the RF algorithm (version 4.6-14) from the R statistical package (version 3.5.3) [60]. The descriptors in those models were analyzed based on the variable importance (permutation test), and the ten most important descriptors from each model were set aside. The last step was an exhaustive search, where unique descriptors that were in the preliminary RF models over five times were used. In this step, only combinations of up to three parameters were used to build MLR models. Finally, the ultimate model was selected among the best models based on the descriptor’s interpretability.

### 2.4. Model Diagnostics and Applicability Domain

The quality of the MLR models was described with the root mean square error (RMSE), the Pearson correlation coefficient for the training set ($R_{train}^{2}$), the cross-validated (leave-one-out) Pearson correlation coefficient ($R_{cv}^{2}$, Equation (1)), and the Pearson correlation coefficient for the validation set ($R_{val}^{2}$),

(1) $$R_{train/cv/val/test}^{2}={\left(\frac{\sum_{i=1}^{n}\left(y_{i}^{calc}-\bar{y_{i}^{calc}}\right)\left(y_{i}^{obs}-\bar{y^{obs}}\right)}{\sqrt{\sum_{i=1}^{n}{\left(y_{i}^{calc}-\bar{y_{i}^{calc}}\right)}^{2}}\sqrt{\sum_{i=1}^{n}{\left(y_{i}^{obs}-\bar{y^{obs}}\right)}^{2}}}\right)}^{2},$$

where, $y_{i}^{obs}$ is experimental $logS_{0}$ value, $y_{i}^{calc}$ is calculated $logS_{0}$ value, $\bar{y^{obs}}$ is the mean value of experimental $logS_{0}$, and $\bar{y^{calc}}$ is the mean value of calculated $logS_{0}$.

In addition, the quality of testing with the tight and loose test sets was described with the RMSE, the measure of prediction performance (MPP), the Pearson correlation coefficient ($R_{test}^{2}$, Equation (1)), and the coefficient of determination for the test set ($R_{det\_test}^{2}$),

(2) $$R_{det\_test}^{2}=1-\frac{\sum_{i}{\left(y_{i}^{obs}-y_{i}^{calc}\right)}^{2}}{\sum_{i}{\left(y_{i}^{obs}-\bar{y^{obs}}\right)}^{2}}.$$

The coefficient of determination was used for the test set, as this describes a correlation, where the slope is one and the intercept is zero. The MPP describes the percentage of predictions that differ from the experimental $logS_{0}$ value by 0.5 units or less [20].

The applicability domain of the QSPR models was analyzed with ranges of descriptor values and Williams plots [61,62]. Williams’ plot is based on each compound’s leverage () and the residual (the difference between the experimental and calculated values). It gives a graphical representation of structurally different and statistically deviating compounds. The leverage value higher than the critical leverage value () shows that the compound can be structurally different, and thus the prediction may have lower accuracy [63,64]. The residuals were used to determine outliers. Strong outliers were defined as compounds with the absolute value of the residual higher than 2 log units. This threshold for strong outliers was also used in the Mitchell’s SC2019 article [25].

### 2.5. Availability of Models

The derived QSPRs together with their source data are provided in the QSAR Data Bank format [65] and have been uploaded to the QsarDB repository [66,67] for exploring, predicting, and independent verification [68]. The presentation of data and models is based on FAIR principles [69].

## 3. Results

### 3.1. The Model for the Training Data Set 1

**Figure 1:**
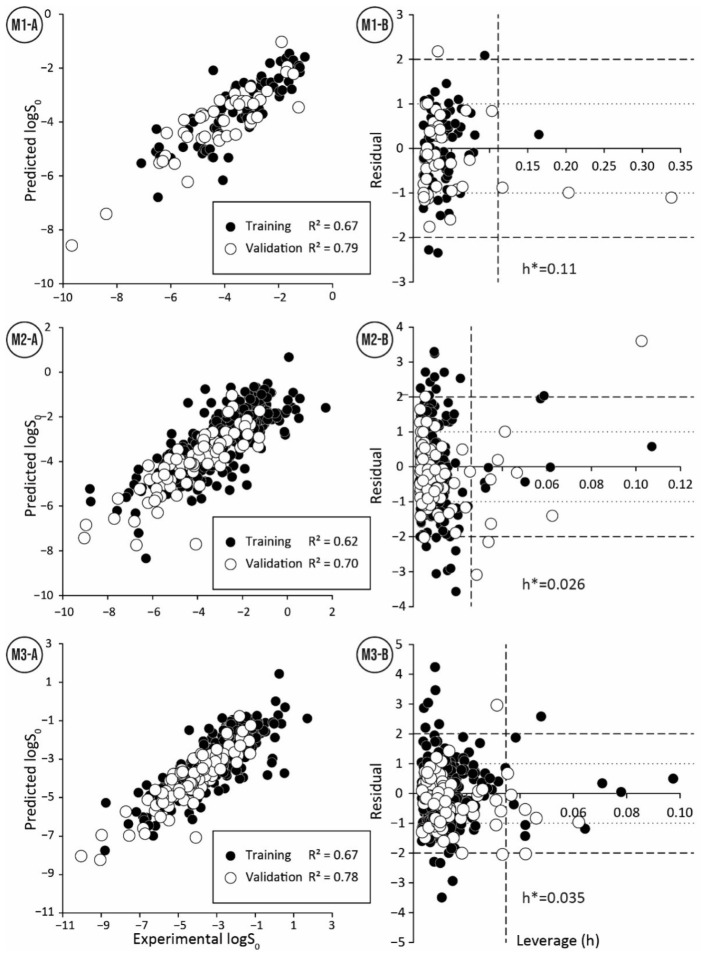
Correlation between experimental and predicted values (**A**) and Williams plots (**B**) for the training and validation set for three QSPR models (**M1**–**M3**).

The QSPR model M1 for the training set with small interlaboratory variability comprised two descriptors (Equation (3), https://doi.org/10.15152/QDB.257, accessed on 19 October 2022). The statistical parameters for the training set ($R_{train}^{2}$ = 0.67) were lower than for the validation set ($R_{val}^{2}$ = 0.79). That can be attributed to three strong outliers (Mefenamic acid, Guanine, and Verapamil) in the training set (Figure 1: M1, Table S1). In contrast, the validation set includes only one strong outlier (Ofloxacin) (Figure 1: M1, Table S1). These outliers can be caused by unreliable experimental measurements or inaccuracies in the calculation of descriptor values (ALOGP2). The small number of strong outliers in the validation set is surprising since compounds among the lowest intrinsic aqueous solubility values were in the validation set (Table 1). This demonstrates the ability of model M1 to extrapolate and predict compounds with very low solubility (Figure 1: M1-A).

(3) $$\begin{array}{c} logS_{0}=8.3304-1.64547\cdot SM04\_EA\left(bo\right)-0.0935641\cdot ALOGP2\\ n_{train}=81,\;R_{train}^{2}=0.67,\;R_{cv}^{2}=0.64,\;RMSE_{train}=0.82,\;n_{val}=42,\;R_{val}^{2}=0.79,\;RMSE_{val}=0.85 \end{array}$$

The descriptor ALOGP2 (squared Ghose–Crippen octanol–water partition coefficient) in the model (Equation (3)) describes the hydrophobicity, which is calculated using the atom contribution method with 90 atom types [70]. The negative coefficient of ALOGP2 shows that more hydrophobic compounds have lower intrinsic aqueous solubility than hydrophilic compounds. The second descriptor in the model (Equation (3)) SM04_EA(bo) (the spectral moment of order 4 from edge adjacency matrix weighted by bond order) describes the presence and position of the heteroatoms via considering bond orders [71]. In addition, this descriptor takes into account the molecular volume, and polarizability or polarity of the molecules present in the pure liquid [72]. All these properties of chemical structure are essential in describing aqueous solubility. SM04_EA(bo) also has the ability to discriminate between isomers, compounds with different branching, the position of heteroatoms, and chain conformation difference [72]. In the case of SM04_EA(bo), it can be concluded that higher intrinsic aqueous solubility is inherent to compounds with fewer multiple bonds (related to bond order) and with more heteroatoms accessible on molecules surface.

### 3.2. Models for Training Data Set 2

Two models (M2 and M3) were derived with different descriptor calculation software and descriptor selection algorithms for the structurally diverse training data set 2. The model M2 was derived from RDKit descriptors by using the OMP descriptor selection algorithm and included two descriptors (Equation (4), https://doi.org/10.15152/QDB.257, accessed on 19 October 2022). The statistical parameters of model M2 shared a similar trend with model M1, where the validation set statistics were higher than the training set statistics. Similarity compared to model M1 was observed also in the number of outliers, where the training set contained more strong outliers than the validation set (Figure 1: M2, Table S2). In addition, like the model M1, the validation set includes compounds with lower solubility than the training set (Table 1).

(4) $$\begin{array}{c} logS_{0}=-0.01662\cdot TPSA-0.84928\cdot MolLogP-0.3583\\ n_{train}=346,\;R_{train}^{2}=0.62,\;R_{cv}^{2}=0.62,\;RMSE_{train}=1.00,\;n_{val}=90,\;R_{val}^{2}=0.69,\;RMSE_{val}=0.98 \end{array}$$

The descriptor TPSA (topological polar surface area) in the model (Equation (4)) describes the size of the molecule and is calculated from the surface areas of polar fragments present in the molecule with the simple group contribution method [73]. This descriptor correlates well with molecular transport properties, such as intestinal absorption and permeation through the blood-brain barrier [73]. The descriptor MolLogP (logarithm of the octanol–water partition coefficient) is calculated using the atomic contribution approach developed by Wildman and Crippen [74]. Since log correlates with aqueous solubility, it has previously been used to estimate solubility. For example, the general solubility equation (GSE) for organic nonelectrolytes [75] includes experimental log and melting point. Additionally, the negative sign of Equation (4) is physically justified, since increasing the lipophilicity leads to a decrease in intrinsic aqueous solubility. MolLogP has the strongest contribution in the model M2, $R^{2}$ values between MolLogP and $logS_{0}$ are 0.54 and 0.61 for the training and validation sets, respectively.

Model M3 (Equation (5), https://doi.org/10.15152/QDB.257, accessed on 19 October 2022) included three descriptors: XlogS, complemented with SpMax1_Bhp (the largest absolute eigenvalue of Burden modified matrix-n1/weighted by relative polarizabilities) and SHBd (Sum of E-States for (strong) hydrogen bond donors). The statistical parameters for model M3 followed the same pattern as previous models. Again, $R^{2}$ value for the validation set was higher than for the training set due to the presence of strong outliers in the training set (Figure 1: M3, Table S3). Compared to two other models, this model has the smallest RMSE for the validation set, but not by a large margin.

(5) $$\begin{array}{c} logS_{0}=0.82617\cdot XlogS-2.71151\cdot SpMax1\_Bhp-0.50016\cdot SHBd+9.14318\\ n_{train}=346,\;R_{train}^{2}=0.67,\;R_{cv}^{2}=0.66,\;RMSE_{train}=0.94,\;n_{val}=90,\;R_{val}^{2}=0.78,\;RMSE_{val}=0.84 \end{array}$$

Descriptor XlogS is derived using a group contribution approach, which includes 83 atom/group types and three correction factors [55]. For the given training set, the XlogS descriptor alone overestimates intrinsic aqueous solubility, particularly for molecules with hydrogen bond donor groups. Therefore, the model needs a descriptor that considers interactions from hydrogen bond donors in the molecule, and this has been taken into account by the descriptor SHBd, which has a negative coefficient as expected. The negative coefficient for the descriptor SpMax1_Bhp implies that an increase in its value decreases solubility. This descriptor characterizes the molecule’s branching and polarizability, and it has been also found that the polarizability of a molecule is among the properties that affect solubility in water [76]. Therefore, it was mechanistically justified to include this descriptor in the model.

### 3.3. Applicability Domain and Outliers

In the context of this study, the applicability domain of models M1-M3 is well-defined by the chemical space of drug substances, as training data sets contain only these types of compounds. Given the complexity of the chemical structure space of drug substances, other approaches describing applicability domain provide additional information when using the models.

**Figure 2:**
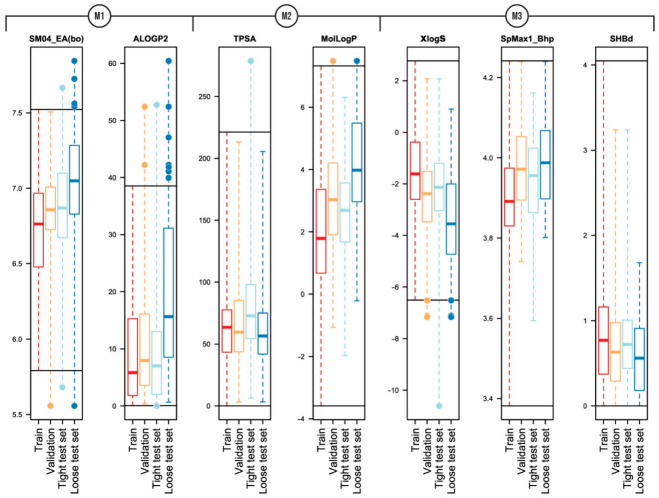
Comparison of descriptor ranges and distributions for training, validation, and test data sets of models (**M1**–**M3**). The horizontal solid line corresponds to the minimum and maximum values of the training data set.

A detailed overview of applicability domain in the context of chemical space can be obtained by comparing the ranges and distribution of molecular descriptor values of the model derived for the training set with the ranges and distributions of the molecular descriptors of the validation and test data sets. A comparison of the boxplots (Figure 2) shows that the distribution of descriptors within the data sets is mostly similar and only a few compounds are outside of descriptor ranges of the training sets. For the model M1, such points exist for both validation and test data series and both descriptors (Figure 2, Table S1). However, compounds with values outside the descriptor ALOGP2 range are usually not strong outliers. For model M2 only three compounds are outside of the descriptor ranges (Table S2). Here, Cyclosporine A with an extreme value for the descriptor TPSA stands out, and this compound is also a strong outlier. For the model M3, compound descriptor values are out of range only for the descriptor XlogS and more than half of them are strong outliers (Table S3).

A consolidated view of the descriptors involved in the QSPR model can be obtained from the analysis of leverage values. A co-analysis of leverage values with residuals, i.e., the Williams plot (Figure 1), makes it possible to identify compounds with high leverage and to further analyze their trends in relation to residuals. Compounds with high leverage value are rarely outliers (see Tables S1–S3, compounds with high leverage are marked), indicating that models M1–M3 can accurately predict the intrinsic aqueous solubility of structurally diverse compounds with one or more extreme descriptor values. The training and validation data sets of models M1–M3 have less than ten compounds with high leverage, showing that the models have a wide applicability domain. For model M1, the descriptor ALOGP2 is the main reason for the high leverage values. However, the probability that compounds with high leverage are also strong outliers increased when the compound’s high leverage value is caused by two extreme descriptor values. For models M2 and M3, most of the high leverage values are due to the combination of several extreme descriptor values. At the extreme values of the XlogS descriptor of the model M3, there is an association between strong outliers and compounds with high leverage.

The analysis of large residuals on the Williams plots shows that strong outliers are usually in the training set and rarely in the validation set (see Tables S1–S3, strong outliers are marked). This may be due to the fact that the training sets include four times more compounds than the validation sets. The quality of training data is also related to the number of outliers in the model. Model M1, based on compounds with a low SD value (i.e., high consensus training data set), includes only three outliers in the training set and one outlier in the validation set. In contrast, for models (M2 and M3) based on compounds with a higher SD value (i.e., structurally diverse training set) there are significantly more outliers: model M2 has 18 strong outliers in the training set and 5 in the validation set, and model M3 has 11 strong outliers in the training set and 5 in the validation set. A comparison of strong outliers in models M1–M3 (Tables S1–S3) indicates that all models had one common strong outlier, Guanine. All models over predict the intrinsic aqueous solubility for Guanine, which may indicate a problematic experimental value. A comparison of outliers in models M2 and M3 indicates eight common outliers in the training set and three in the validation set. Considering that different modeling approaches do not affect the prediction of these molecules, it suggests inaccuracies in their experimental values or the presence of structural features that cannot be properly characterized by used molecular descriptors.

### 3.4. Prediction of SC2019 Test Sets

**Figure 3:**
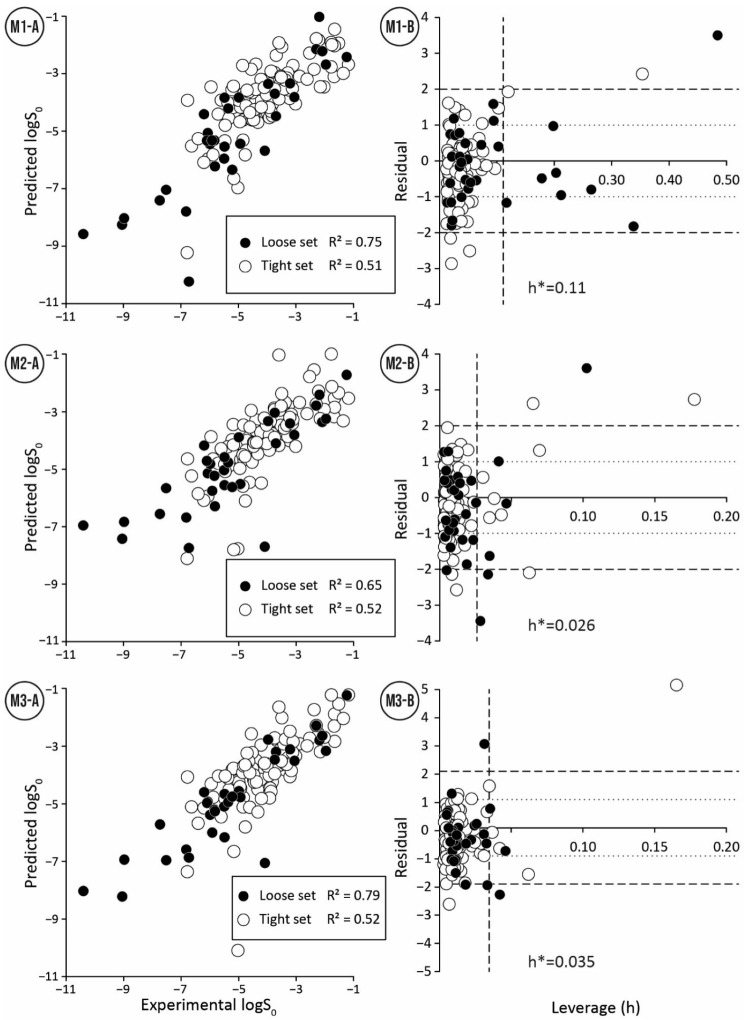
Relationship between experimental and predicted values (**A**) and Williams plots (**B**) for tight and loose test sets made by three QSPR models (**M1**–**M3**).

Models M1-M3 show comparable prediction capability (Table 2) for the SC2019 tight test set: ($R_{test}^{2}$~0.5, $R_{\det\_test}^{2}\;$0.38 … 0.45, RMSE 0.94 … 0.99, MPP 42 … 48%). The prediction quality for the SC2019 loose test set was expected to be lower than the tight test set because the experimental data in the tight test set can be considered more accurate due to lower average inter-laboratory standard deviation (~0.15 vs. ~0.62). However, it turned out opposite, and for the loose test set, models M1-M3 have a higher correlation between predicted and experimental $logS_{0}$ values ($R_{test}^{2}$ 0.65 … 0.79, $R_{\det\_test}^{2}$ 0.62 … 0.75) compared to the tight test set. At the same time, the RMSE values (1.06 … 1.32) are higher and MPP (31…44%) values lower than for the tight test set (Table 2, Figure 3). A possible explanation could be a wider range of experimental $logS_{0}$ values (5.6 vs. 9.16 units) for the loose test set. In this case, the correlation between experimental and calculated values is less influenced by the experimental errors. On the other hand, the loose test set contains more compounds with low solubility values, which are more difficult to measure, and their predictions have larger errors. Therefore, RMSE and MPP have lower values for the loose test set compared to the tight test set as expected.

To simplify the comparison of developed models and to have a more realistic estimation about the distribution of prediction errors, the tight and loose test sets were merged. As expected, all statistical parameters in the merged test set were within the statistical parameters of the tight and loose test sets considered separately (Table 2). All three models M1–M3 also showed even more similar prediction capability for the merged test set than for separate test sets (Table 2: $R_{test}^{2}$ 0.6 … 0.65, $R_{\det\_test}^{2}$ 0.58 … 0.61, RMSE 1.00 … 1.05, MPP 43 … 45%).

The quality of prediction statistics for both test sets is highly influenced by strong outliers (> ±2 $logS_{0}$ units, Figure 3, Tables S1–S3). Eliminating strong outliers significantly improved the correlations between experimental and predicted values for the tight test set ($R_{test}^{2}$~0.6, $R_{\det\_test}^{2}$ 0.51 … 0.60) and the loose test set ($R_{test}^{2}$~ 0.81 … 0.89, $R_{\det\_test}^{2}$ 0.78 …0.86). In the tight test set, three frequent outliers (Table 3) are Cisapride (in M1, M2, M3), Folic acid (in M1, M2), and Cyclosporine A (in M2, M3). Three frequent outliers (Table 3) in the loose test set were Amiodarone (in M2, M3), Itraconazole (in M2, M3), and Rifabutin (in M2, M3). The structural features common to these outliers are the size and complexity characterized by a high number of cycles and functional groups, respectively. It has been shown before that for very large compounds (molecular mass over 500), it is more complicated to predict the intrinsic aqueous solubility than for smaller compounds [77,78]. The training set for the model M1 includes only one very large compound (Dipyridamole, molecular mass > 500), while models M2 and M3 include seven such compounds (Paclitaxel, Erythromycin, Trilazad, Tipranavir, SB209670, Levothyroxine, Dipyridamole). Nevertheless, the test sets contained 15 very large compounds, and the models can still predict most of them with high accuracy. The wide applicability domain of models M1-M3 is confirmed both by the Williams plot (Figure 3), where very few compounds have high leverage values, and by the descriptor values, where majority of the compounds of the test data sets are in descriptor ranges of the training data sets (Figure 2).

### 3.5. Consensus of Models

The predictions for the SC2019 test sets (see previous chapter) show that the models M1-M3 perform similarly considering the overall statistical metrics (Table 2) but have notable differences in handling outliers. This led to a consensus approach to average the predicted values from all three models. The consensus approach can have additional advantages because all three models were developed with different workflows starting from different training sets, different molecular descriptor calculation software, and molecular descriptor selection algorithms (Table 1). Considering all statistical parameters, the consensus of models improved prediction accuracy for SC2019 test sets relative to the individual models (Table 2: M_cons, https://doi.org/10.15152/QDB.257, accessed on 19 October 2022). The improvement was more pronounced for the tight test set. Looking at the merged test set revealed that individual models had 13 strong outliers. After the consensus prediction (Table S4), only six strong outliers remained. Consequently, the consensus of models decreased the probability of finding strong outliers and prediction errors in general.

### 3.6. Comparison with the Solubility Challenge 2019

An analysis of the predictions of the different models in the SC2019 summary article showed that the predictions of our MLR models were among the best in their category [24]. An additional comparison with the best SC2019 models was performed for models M1-M3 and M_cons. For this purpose, models with the best statistical parameters ($R^{2}$, $R_{\det\_\mathrm{test}}^{2}$, RMSE, and MPP) for the predictions of tight and loose test sets were selected from the SC2019 results [24]. This included only models that had all performance statistics in the top 20. Whereas in SC2019 [24] the analysis of the prediction results was performed separately for tight and loose test sets, here both test sets were analyzed together. The aim of this approach was to simplify the comparison of models and to have a more realistic estimation of prediction errors by including measurements with high and low experimental SD. Although 12 models qualified for comparison, only 10 models were selected, in addition to models M1, M2, M3, and M_cons. Two models (YUMPU and SGURV) were left out because their training sets also included test set compounds and therefore did not meet the conditions of SC2019. Predictions from the Random Forest regression model of Avdeef [20] were also included in the comparison, with which the intrinsic aqueous solubility values of the SC2019 test sets were also published. The predictions of this model did not participate in SC2019, since the author of the article was one of the initiators of the challenge. In the SC2019 summary article, our MLR models (M1, M2, and M3) are labeled as UMUT-A, UTUM-B, and UTUM-C, respectively [24].

**Figure 4:**
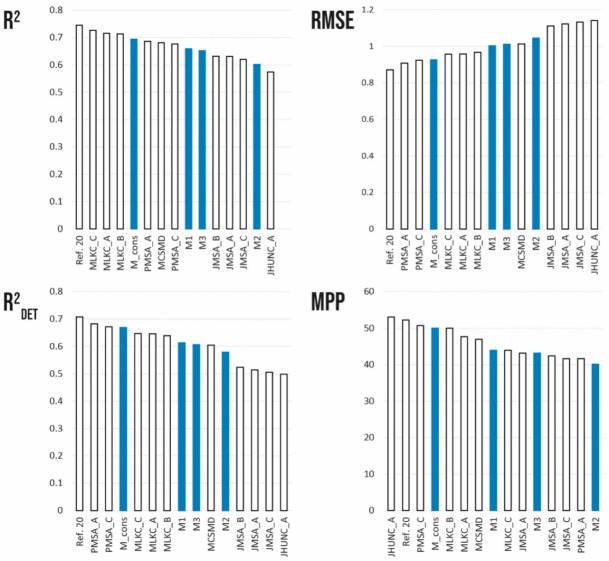
Comparison of Solubility Challenge 2019-submitted best models, consensus model (M_cons) published in this paper, and previously published RF model ([20]). Models from present study are marked as blue.

The best models selected differed in terms of modeling methods, size of training sets, and the number of descriptors in the model (Table 4). A comparison of the four statistical parameters indicated that all models have similar prediction capabilities for the merged test set (Figure 4). For example, the MPP of the selected 17 models does not exceed 60% for any of the models, so all models have a medium predictability of intrinsic aqueous solubility, and they show very similar statistical parameters (Figure 4: MPP). In the comparison of the performance parameters of all modeling methods used in SC2019, models M1-M3 are placed in the middle of the lineup of top models, while the consensus of the models (M_cons) is always in the first third of the lineups.

## 4. Discussion

In order to improve the result of intrinsic aqueous solubility modeling, all the development stages of QSPR need attention, which can be grouped as follows: the input (training) data, the suitability of descriptors, the model development methods, and analyzing the limits of models (outliers). The following subchapters discuss these issues based on the decisions made in this research.

### 4.1. Fit-For-Purpose Training Set

In data-driven modelling, the content and quality of the training set determine the predictive quality and applicability domain of the model. In SC2019, no common training data set was provided, and all participants had to either use an existing model or create their own training set. Therefore, the basis of modeling for us was the design and construction of the fit-for-purpose training set that would be optimal for predicting the intrinsic aqueous solubility of the given test sets. Since the test sets provided by SC2019 targeted drug substances, the main goal of the compilation of training data was to target the chemical space of drug substances and the quality of experimental values. Two approaches were focused on. In the first approach, only high-quality data was used (model M1), and in the second approach, a larger but noisier data set was curated (models M2 and M3). The QSPRs developed on these two data sets gave similar results for the prediction of compounds in the test set (Table 2), indicating that correctly targeted training data is a prerequisite for prediction quality and can be achieved even when training on small data sets. The same conclusion can be drawn for the SC2019 results as a whole, where QSPRs on much larger data sets (over 1000 compounds) yielded similar prediction accuracy to smaller training sets of only ~100 compounds (Table 4, Figure 4).

### 4.2. Molecular Descriptors and Their Relevance

Software for calculating molecular descriptors differ in the number and types of descriptors. Differences may also be due to the algorithms and implementation details. This affects the quality of the developed QSPR models and their predictive ability. In this study, different descriptor calculation software (Dragon, RDKit, PaDEL-Descriptors, XLogS) were used to derive different models, but all models included descriptors that characterize similar features of molecular structures, such as hydrophobicity and polarity. Looking at the prediction models submitted for SC2019, they used an even wider selection of descriptor calculation programs, but none of them performed significantly better than the others within the derived models. In addition, the relevance and number of descriptors included in the model should also be reviewed. Current descriptor selection algorithms only selected descriptors important for the dissolution process, so there were only two or three descriptors in the models M1-M3. The best models submitted to SC2019 contain more than 50 descriptors (Table 4). The large number of descriptors in the model makes their relevance analysis difficult and may lead to overfitting. Interestingly, the models with a large number of descriptors do not have significantly better prediction performance when compared to the models with a smaller number of descriptors.

The analysis of the molecular descriptors in models M1-M3 based on the relevance to the solubility process shows that selected molecular descriptors are related to the three key molecular transition interactions from the crystalline state into an aqueous solution [76,79,80,81,82]: (i) dissociation of the molecule from the crystal, (ii) formation of a cavity in the solvent, and (iii) insertion of the molecule into the solvent. The dissociation of the molecule from the crystal depends on the intermolecular interactions between the molecules in the crystal lattice and is usually related to the melting point [75]. In models M1-M3, this interaction is described with polarity/polarizability-related descriptors, such as SM04_EA(bo) (M1), TPSA (M2), SpMax1_Bhp, and SHBd (M3). The polarity/polarizability is also reflecting intramolecular forces and interactions between molecules. The formation of a cavity in the solvent is related to the surface area of the molecule [13,14], which in its simplest form is described with molecular weight [76]. This effect is usually indirectly included into polarity/polarizability-related descriptors through the volume and polarizability (SM04_EA(bo)), presence of polar atoms in a molecule (TPSA), and polarity (SpMax1_Bhp*)* or hydrophobicity (different $logP_{ow}$-s). Hydration is commonly related to hydrophobicity ($logP_{ow}$*)*, directly included in models M1 (ALOGP2) and M2 (MolLogP). Thus, it can be concluded that the molecular descriptors in the models are consistent with the components of the solubility process, and the derived models thus have a mechanistic explanation.

### 4.3. Selection of a Method for Model Development

The use of complex machine learning and artificial intelligence methods in the development of QSPR models is currently very trendy, and therefore it was suspected that many of the predictions made for SC2019 would use these types of models. Different machine learning methods (RF, support vector machine (SVR), MLR) were also tested on training data set 2 to select an appropriate method for developing QSPRs. The MLR method produced models with similar statistical parameters compared to the models obtained by the RF and SVR methods. A decision in favor of MLR was also based on the ease of interpretation of the models and the mechanistic insights they can provide. As it turned out, most of the predictions submitted to SC2019 were made with models based on complex methods (mainly neural networks) [24] and the predictions of these complex models were not significantly better than the predictions of MLR models.

Although the predictive performance of the MLR models was good, efforts were made to improve it by exploring a consensus approach. Combining models developed with different training sets, different descriptor calculation programs, and model development methods proved beneficial as it further improved predictive performance. The consensus approach was also unaffected by strong outliers in one model, while the predictions of the other two models proved to be accurate. For the same reason, many of the predictions made for SC2019 have been obtained with consensus models, especially among the top models (e.g., consensus of different ML methods, RFR models, light gradient boosting machines, etc.). In conclusion, the prediction results of the models derived for small, high-quality data sets were also improved by the consensus of model predictions in the assessment of intrinsic aqueous solubility, being an alternative to models derived for large data sets, where the solubility at the property level is not clearly determined.

### 4.4. Outliers in the SC2019 Test Sets

While a thorough outlier analysis was performed for the Solubility Challenge 2008 [18,19], one was not performed for the SC2019 [24]. To fill this gap, predictions from 15 models were selected from SC2019 (see Section 3.6) and the six most frequent strong outliers (±2 $logS_{0}$ units) were identified. In the tight test set Cisapride and Folic acid were two outliers in 12 and 13 models out of 15, respectively. In the loose test set Amiodarone, Clofazimine, Itraconazole, and Pioglitazone were four outliers in 14, 9, 12 and 8 models, respectively. Four of these six compounds are strong outliers in at least two models M1, M2, or M3 (Table 3). Such systematic outliers may be due to questionable intrinsic aqueous solubility values.

A thorough analysis of the outliers shows that the intrinsic aqueous solubility of all of them is overestimated. Mean solubility is given in SC2019 for Amiodarone, Clofazimine, Itraconazole, and Pioglitazone [20]. The experimental data for each of these drug substances vary widely (over one unit) and the standard deviation for each experimental value is high (above 0.5 units). Among these, the most problematic compound is Amiodarone, as almost all the selected models mispredict its solubility, suggesting potential problems with the experimental value. Specifically, the reported $logS_{0}$ values for Amiodarone (−9.68 … −11.06 and SD 0.59 … 0.97 [20]) indicate a high variance with a large standard deviation, which means that the experimental intrinsic aqueous solubility value may be incorrect. Using the highest experimental $logS_{0}$ value as a reference, Amiodarone is an outlier in fewer models, 11 out of 15. It follows that the choice of experimental reference values is an important factor in the development of models and analyzing the predictive capability of models.

Another explanation could be that Amiodarone has the lowest intrinsic aqueous solubility value in the loose test set (−10.4) together with two other strong outliers, Clofazimine (−9.05) and Itraconazole (−8.98). These compounds are extremely low soluble and there is a high probability that the training set for most of the models of solubility used to provide predictions may not include compounds with extremely low intrinsic aqueous solubility values. For example, such compounds may be problematic for decision tree and random forest methods, as these methods cannot extrapolate outside the property range of their training sets. It should be highlighted that these four compounds (Amiodarone, Clofazimine, Itraconazole, and Pioglitazone) of the loose test set are not outliers in model M1 predictions, although some of them are outliers in model M2 and M3 predictions. This suggests that a model developed on a small set of high-quality data can provide more reliable extrapolation than a model developed on a large set of data but of lower quality. Additionally, it also highlights that the training data set must include low soluble compounds to improve the prediction quality of very low soluble compounds.

## 5. Conclusions

The presented research allows us to conclude that the fit-for-purpose training data makes it possible to derive straightforward multi-linear regression models that allow mechanistically transparent interpretations and successful prediction of the intrinsic aqueous solubility of drug substances, and that a consensus approach made it possible for the data-driven QSPR model predictions to complement each other.

All three derived MLR models perform well individually while being mechanistically transparent and easy to understand. All molecular descriptors involved in these models are related to the following key steps in the solubility process: dissociation of the molecule from the crystal, formation of a cavity in the solvent, and insertion of the molecule into the solvent. The consensus model of these three MLR models remarkably improved prediction capability by reducing the number of strong outliers more than two times.

These models have potential applications in drug discovery and development stages, because the training sets are diverse and focused on drug substances. The predictive ability of models has been successfully evaluated with blind testing on data sets provided in the frame of the SC2019 initiative. While all individual models (M1, M2, M3) performed well, the consensus model showed the best performance in all model evaluation metrics. All developed models have been published [68] in the QsarDB repository according to FAIR principles [69] and can be freely explored, downloaded, and most importantly, used for predictions.

The retrospective analysis of the best predictions from the SC2019 initiative [24] has been carried out in two aspects that have not been covered before. The first analysis covered outliers and indicated that the cause of some strong outliers may be related to potentially incorrect experimental values rather than the modeling method. The second analysis investigated the performance of models on merged test set to have a more realistic estimation of the distribution of prediction errors. This combined analysis indicated that most of the predictions submitted to SC2019 are performing similarly, and no significant differences can be detected.

## Figures and Tables

**Table 1:** Overview of the structure of the methodology.

	M1	M2	M3
Data set type	Small high-quality	Large high-variety compounds	
Training/validation/test	81/42/132	346/90/132	
Range of $logS_{0}$ in training set (average, SD)	−7.1 … −1.03 (−3.55, 1.43)	−8.8 … 1.7 (−3.14, 1.64)	
Range of $logS_{0}$ in validation set (average, SD)	−9.68 … −1.27 (−4.18, 1.77)	−10.05 … −1.24 (−4.29, 1.74)	
Range of $logS_{0}$ in test set (average, SD)	−10.4 … −1.18 (−4.32, 1.62)		
Tight test set	−6.79 … −1.18 (−4.03, 1.27)		
Loose test set	−10.4 … −1.24 (−5.24, 2.18)		
Descriptor calculators	Dragon	RDKit	XLOGS, PaDEL-Descriptors
Model development software	CODESSA Pro	scikit-learn	R statistical package
Descriptor selection approach	Stepwise forward selection	Orthogonal matching pursuit	Based on most common descriptors in RF models

**Table 2:** Statistical parameters for the tight and loose test set predictions of models M1–M3.

	Model	M1	M2	M3	M_cons
Tight test set	$R_{test}^{2}$	0.51	0.52	0.52	0.57
	$R_{\det\_test}^{2}$	0.42	0.45	0.38	0.51
	RMSE	0.97	0.94	0.99	0.89
	MPP	48%	42%	43%	54%
	Nr. of strong outliers	4	5	2	4
Loose test set	$R_{test}^{2}$	0.75	0.65	0.79	0.79
	$R_{\det\_test}^{2}$	0.74	0.62	0.75	0.77
	RMSE	1.1	1.32	1.06	1.04
	MPP	31%	34%	44%	38%
	Nr. of strong outliers	1	3	3	2
Tight and loose test set together	$R_{test}^{2}$	0.66	0.60	0.65	0.69
	$R_{\det\_test}^{2}$	0.61	0.58	0.61	0.67
	RMSE	1.00	1.05	1.01	0.93
	MPP	44%	40%	43%	50%
	Nr. of strong outliers	5	8	5	6

**Table 3:** Strong outliers (±$logS_{0}$2 units) in the test sets for at least two models.

Tight Set		
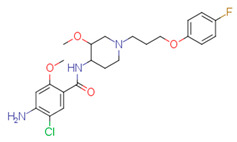 *Cisapride* Experimental: −6.78 M1: −3.92 M2: −4.64 M3: −4.07 M_cons: −4.21	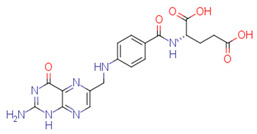 *Folic acid* Experimental: −5.96 M1: −3.45 M2: −3.87 M3: −4.31 M_cons: −3.88	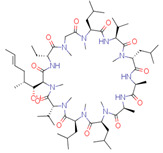 *Cyclosporine A* Experimental: −5.03 M1: −6.96 M2: −7.77 M3: −10.09 M_cons: −8.27
**Loose set**		
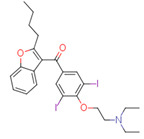 *Amiodarone* Experimental: −10.4 M1: −8.58 M2: −6.96 M3: −8.03 M_cons: −7.86	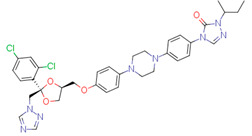 *Itraconazole* Experimental: −8.98 M1: −8.03 M2: −6.84 M3: −6.94 M_cons: −7.27	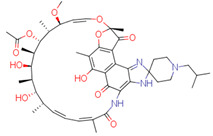 *Rifabutin* Experimental: −4.09 M1: −5.68 M2: −7.70 M3: −7.06 M_cons: −6.81

**Table 4:** Size of training sets and number of descriptors in the top SC2019 models *^#^.

	Small Training Set (<500 comp.)	Large Training Set (>500 comp.)
Many descriptors (>50)	JMSA_A (117/73/Cons. MLR) JMSA_B (117/73/extra tree reg.) JMSA_C (117/73/RFR) JHUNC_A (312/NA/ANN)	PMSA_A (2220/168/RBF) PMSA_C (7841/164, RBF) MLKC_A-C (881/NA/light GMB) *RF* [20] *(4449/NA/RFR)*
Fewer descriptors (<50)	M1 (UMUT_A, 81/2/MLR) M2 (UMUT_B, 346/2/MLR) M3 (UMUT_C, 346/3/MLR) M_cons (346 + 81, 7, consensus)	MCSMD (2666/7/ANN)

* description of model name: model name in SC2019 (number of compounds in training set/number of descriptors in model/used method for modeling). ^#^ Models not submitted to Solubility Challenge 2019 are in italic.

## Data Availability

The data presented in this study are openly available in QsarDB.org repository at https://doi.org/10.15152/QDB.257, reference number QDB.257.

## Supplementary Materials

The following supporting information can be downloaded at: https://www.mdpi.com/article/10.3390/pharmaceutics14102248/s1, Table S1: Data for model M1. Table S2: Data for model M2. Table S3: Data for model M3. Table S4: Data for model M_cons.
